# Targeting the oncogenic transcription factor FOXM1 to improve outcomes in all subtypes of breast cancer

**DOI:** 10.1186/s13058-023-01675-8

**Published:** 2023-06-27

**Authors:** Benita S. Katzenellenbogen, Valeria Sanabria Guillen, John A. Katzenellenbogen

**Affiliations:** 1 Department of Molecular and Integrative Physiology, University of Illinois at Urbana-Champaign, Urbana, IL 61801 USA; 2 Cancer Center at Illinois, University of Illinois at Urbana-Champaign, Urbana, IL 61801 USA; 3 Institute for Genomic Biology, University of Illinois at Urbana-Champaign, Urbana, IL 61801 USA; 4 Department of Chemistry, University of Illinois at Urbana-Champaign, Urbana, IL 61801 USA

**Keywords:** FOXM1, Breast cancer, Inhibitors, Transcriptional activity, Drug resistance

## Abstract

FOXM1 (Forkhead box M1) is an oncogenic transcription factor that is greatly upregulated in breast cancer and many other cancers where it promotes tumorigenesis, and cancer growth and progression. It is expressed in all subtypes of breast cancer and is the factor most associated with risk of poor patient survival, especially so in triple negative breast cancer (TNBC). Thus, new approaches to inhibiting FOXM1 and its activities, and combination therapies utilizing FOXM1 inhibitors in conjunction with known cancer drugs that work together synergistically, could improve cancer treatment outcomes. Targeting FOXM1 might prove especially beneficial in TNBC where few targeted therapies currently exist, and also in suppressing recurrent advanced estrogen receptor (ER)-positive and HER2-positive breast cancers for which treatments with ER or HER2 targeted therapies that were effective initially are no longer beneficial. We present these perspectives and future directions in the context of what is known about FOXM1, its regulation, and its key roles in promoting cancer aggressiveness and metastasis, while being absent or very low in most normal non-regenerating adult tissues. We discuss new inhibitors of FOXM1 and highlight FOXM1 as an attractive target for controlling drug-resistant and difficult-to-suppress breast cancers, and how blocking FOXM1 might improve outcomes for patients with all subtypes of breast cancer.

## Breast cancer is highly heterogeneous but FOXM1 is expressed in all subtypes

Breast cancer is the most common cancer in women worldwide, and its incidence has been increasing due to growth of the aging population [1], and emergent trends in lifestyle factors including obesity, physical inactivity, and alcohol use [2]. The nature of the breast tumors also varies by ethnicity, with African American women having the highest rate of the most difficult to treat triple negative breast cancer (TNBC) subtype compared with other ethnic groups in the United States [3].

A major challenge in treating breast cancer effectively is the very heterogeneous nature of the disease, which encompasses a wide array of histological and molecular characteristics [1, 3, 4] that divides broadly into 6 intrinsic subtypes: Normal-like, Basal-like, Claudin-low, human epidermal growth factor 2 (HER2)-enriched, Luminal B, and Luminal A. While estrogen receptor (ER)-targeted and HER2-targeted therapies are subtype-specific, and are highly effective as first-line treatments [5–7], the efficacy of these therapies can change over time, becoming less effective with treatment and disease progression [6]. Since TNBC lacks druggable targets like ER, progesterone receptor or HER2, the majority of TNBC patients receive a chemotherapy regimen [8], which can be effective, but may cause more acute and chronic toxicities [9].

The heterogeneous nature of breast cancer and especially advanced breast cancers, and loss, mutation or other alterations of treatable markers such as ER or HER2 over time present significant challenges in finding effective diagnostic, prognostic, and therapeutic strategies [10]. Thus, new targeted therapies are needed both for the treatment of some primary breast cancers and for advanced, treatment-resistant, and metastatic breast cancers. Herein, we discuss new approaches to targeting the oncogenic protein FOXM1 including new inhibitors blocking its many activities. Because FOXM1 is expressed at all breast cancer stages and in all breast cancer subtypes, FOXM1-targeted therapies hold promise for suppressing early breast cancers as well as advanced drug-resistant forms of breast cancers. Further, because the level of FOXM1 generally increases as breast tumors progress, it suggests that the benefit from targeting FOXM1 might actually increase at later stages of the disease.

## FOXM1 as a new target for effective treatment of aggressive breast cancers

FOXM1 is a key regulator of the cell cycle with important roles in many stages, including the G1/S phase transition, entry into mitosis, and proper execution of mitosis [11]. FOXM1 is ubiquitously expressed in the growing embryo, and *foxm1* knockout in mice is lethal due to major developmental defects [11]. During normal development, FOXM1 is a critical regulator of mammary gland morphogenesis and luminal epithelial cell fate by repressing expression of GATA3, a critical regulator of luminal differentiation [12, 13]. The studies of Carr et al. [12] suggest that the ability of FOXM1 to promote the expansion of undifferentiated mammary cells may contribute in mammary tumor development under activating conditions. Aberrant, elevated expression of FOXM1, which is observed in breast tumors, is associated with increased proliferation, invasion and metastasis, and poor patient outcome [14–17]. Because this review is focused on new understanding of FOXM1 and its role in the aggressiveness of many breast cancers, and on the development of inhibitors targeting FOXM1, we refer readers to very thorough earlier reviews on FOXM1 activities during mammary gland morphogenesis [11–13].

In adults, FOXM1 is detectable in self-regenerating tissues such as the liver, where it is activated by injury [18]. Thus, FOXM1 is not expressed in quiescent or differentiated cells, but is expressed in a few proliferating tissues such as testis, and its expression in adults is activated in response to proliferation signals including growth factors or hormones when cells re-enter the cell cycle, as is the case in cancer [19]. Notably, FOXM1 is upregulated and overexpressed in many aggressive ER-positive, HER2-positive, and TNBCs. Hence, therapy directed at FOXM1 in adults with cancer is likely to have an effect principally on cancer cells rather than normal cells, as discussed in more detail later in the Summary and Future Perspectives Section.

## FOXM1 protein and functional domains

**Fig. 1:**
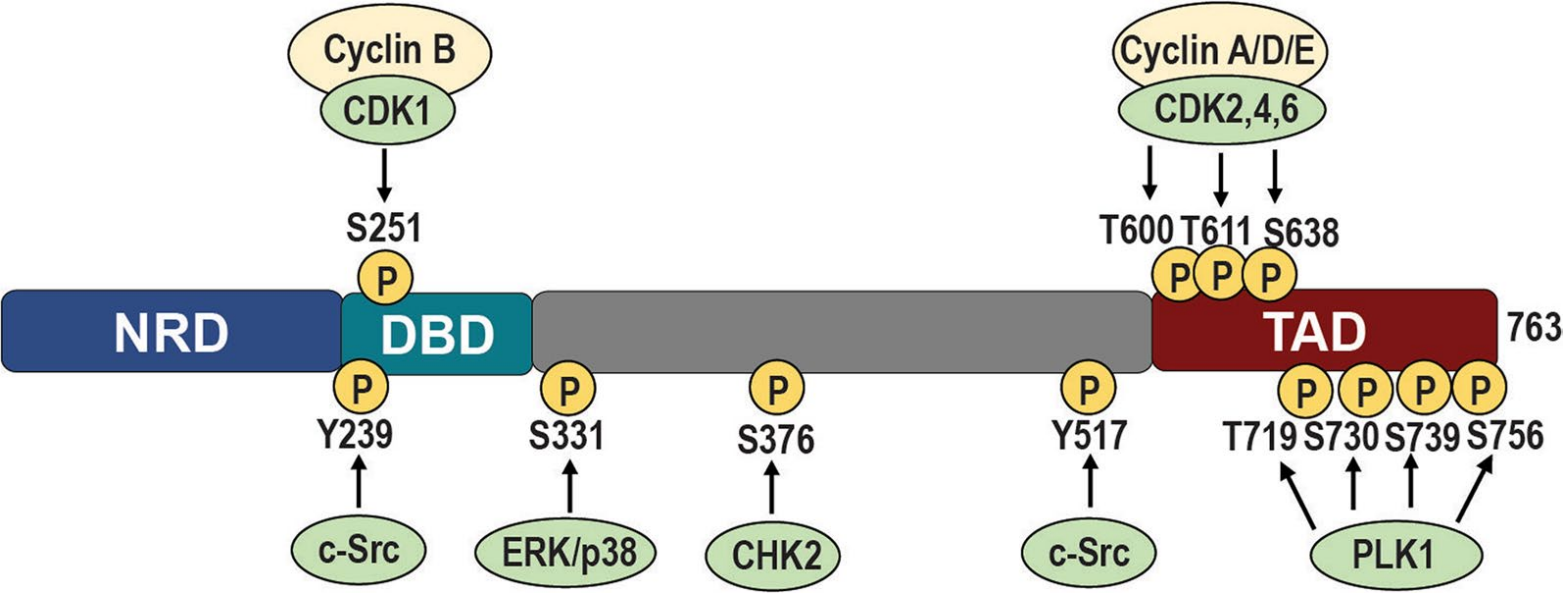
FOXM1 protein domains and phosphorylation sites. Schematic showing the domain structure of human FOXM1c (763 amino acids), its phosphorylation sites and the protein kinases involved. Phosphorylation of FOXM1 activates FOXM1 nuclear translocation and transcriptional activity, and is crucial to relieve autorepression by the N-terminal Repressor Domain (NRD). See text for further details. *NRD* N-terminal Repressor Domain, *DBD* DNA binding (forkhead) domain, *TAD* Transactivation Domain

The FOXM1 protein contains several functional domains, comprised of an N-terminal negative regulatory domain (NRD), the iconic DNA binding Forkhead box domain (DBD), and a C-terminal transcriptional activation domain (TAD), the latter two being linked by a central structural domain [20–23] (Fig. 1). The human FOXM1 gene contains 10 exons, and alternative splicing of two of the exons (A1 and A2) produces four FOXM1 isoforms, FOXM1a, FOXM1b, FOXM1c, and FOXM1d, that differ in the TAD and have different activities [17]. FOXM1a, the longest at 801aa, contains regions encoded by exons A1 and A2 and binds to DNA but lacks transcriptional activity. FOXM1b (748aa) contains neither exon, and FOXM1c (763aa) contains only exon A1 encoding a 15 amino acid insertion region. FOXM1b and FOXM1c have 98% amino acid identity and both are transcriptionally active [17]. The more recently discovered isoform FOXM1d (786aa) contains exon A2 but not exon A1 and appears to be cytoplasmically localized and to act through a non-transcriptional mechanism involving direct interaction with Rho-associated kinase 1/2 [23]. See reference 17 for a schematic of the FOXM1 gene structure and the splicings that generate the four FOXM1 isoforms. While the FOXM1b and FOXM1c isoforms share similar levels of transcriptional activity, analysis of 32 TCGA cancer types and many GTEx normal tissues showed that FOXM1c is the most highly expressed isoform in both contexts [14].

The FOXM1 DBD binds DNA sequences containing tandem repeats of the consensus sequence TAAACA [24] and FOXM1 also interacts with target genes by binding to non-consensus sequences through a process facilitated by its tethering to partner proteins like MYB and MuvB [25]. The function of the TAD is to recruit coregulatory factors and transcriptional machinery to regulate the transcription of target genes. The FOXM1 NRD is an autoregulatory region that associates with the TAD to prevent its association with factors like CBP and p300, repressing FOXM1 transcriptional activity [26]. Autorepression by the NRD is lifted following key signals, such as phosphorylation of the TAD domain by G2/M transition polo-like kinase 1 (PLK1) or cyclin dependent kinases, and additionally by c-Src and p38/MAPK [26–28] (see Fig. 1). Recent structural studies reveal that the NRD and TAD are intrinsically disordered domains that, upon phosphorylation, adopt a structured conformation which allows interaction with CBP and other partner proteins enabling activation of target gene transcriptions [27].

## Activation of FOXM1 by key phosphorylations regulates critical transitions of the cell cycle

Coordination of the various functions of FOXM1 throughout the cell cycle involves initial induction of FOXM1 expression, followed by different rounds of site-specific phosphorylations that regulate its subcellular distribution, interaction partners, and patterns of gene regulation. In response to growth stimuli, FOXM1 expression is induced during G1-phase through activation of its promoter by other transcription factors such as c-Myc [17]. FOXM1 mRNA levels peak during G1/S, but its transcriptional activity is relatively low during this phase due to its cytoplasmic localization and inhibition by its NRD [17]. In late G1, Cyclin D-CDK4/6 complexes phosphorylate multiple sites in the FOXM1 C-terminus, stabilizing the protein and stimulating its transcriptional activity to drive the expression of genes important in the G1/S transition, such as CCNE1 [20]. FOXM1 is also a substrate of the tyrosine kinase c-Src (Fig. 1), and recent work has shown that mutation of either of two critical tyrosines (Y239, Y517) prevents these phosphorylations and FOXM1 nuclear translocation, and greatly impairs FOXM1 activity, breast tumorigenesis, and cancer progression in a luminal B breast cancer model [28].

Phosphorylation of FOXM1 via the Raf/MEK/MAPK pathway also stimulates FOXM1 nuclear translocation and transcriptional activity in late S phase, as well as in G2/M [16]. FOXM1 also helps to advance cell entry into S phase by suppressing nuclear levels of inhibitory cell cycle regulators p21^Cip1^ and p27^Kip1^ [29]. Another important role of FOXM1 in checkpoint control and DNA repair involves its phosphorylation by Chk2 in response to DNA damage and stimulation of the expression of DNA repair genes [30]. FOXM1 is involved in mediating the S/G2 transition through coupling the end of S phase to CDK1-mediated phosphorylation of FOXM1 and subsequent transactivation of the mitotic gene network after ATR de-repression [31]. During the G2 phase, cyclin A/E-CDK2 complexes phosphorylate FOXM1 at several sites including T600, T611, and S638, which relieves repression of the TAD by the NRD and restores FOXM1 transactivation activity [20, 26]. In G2/M, FOXM1 activates transcription of CDC25B, which is required for activation of the Cdk1-Cyclin B complex, and FOXM1 also activates essential G2/M targets including Aurora kinase B (AURKB), Cyclin B1 (CCNB1), and PLK1 through direct interaction with their promoters [16, 32].

FOXM1 also plays a key role in proper mitotic spindle checkpoint function and chromosome stability through transcriptional activation of centromere protein-F (CENP-F) and AURKB, and it regulates several genes that are essential for chromosome segregation and mitosis, including Nek2, KIF20A, and CENP-A [33, 34]. Consistent with all of these roles, ablation of FOXM1 protein expression results in polyploidy, centrosome amplification, mitotic spindle defects, and ultimately, mitotic catastrophe [34, 35]. FOXM1 reaches hyper-phosphorylation and peak activity in M phase, and is dephosphorylated in late M phase, coinciding with exit from mitosis [36]. To end the mitotic gene expression program, the FOXM1 protein is ubiquitinated and rapidly degraded by the proteasome at the onset of anaphase through direct interaction with CDH1 [36].

## FOXM1 overexpression and cancer prognosis, aggressiveness, and metastasis

The overexpression of FOXM1 in many human cancers is associated with advanced tumor stage, high proliferation rate, tumor aneuploidy, and poor prognosis [14, 37–39]. Elevated FOXM1 is correlated with adverse outcome across 39 solid human malignancies, and its overexpression was significantly associated with worse 3-year and 10-year overall survival [40, 41]. Comparison of 32 TCGA cancer types and matched normal tissues showed that FOXM1 mRNA was overexpressed in all cancer types compared to normal tissues, and that FOXM1 mRNA and protein level were highly correlated in all of these cancers [14]. The FOXM1 gene was the top gene most frequently associated with adverse risk in a pan-cancer analysis of 18,000 human tumors, and it outperformed MK167, which encodes the clinically used proliferation marker Ki-67 [40]. FOXM1 is now used in the clinic as a prognostic and predictive marker for stage and grade of bladder cancer [42], and has been identified as the most significant prognostic factor for overall survival in patients with hepatocellular carcinoma [40, 41].

That FOXM1 is an aggressiveness factor in human breast cancers is increasingly well established [37–39, 43]. Of note, primary breast cancers of all subtypes express FOXM1 protein and mRNA at much higher levels compared to normal breast tissue [14, 39], and analysis of matched breast tumor and normal breast tissue pairs showed that FOXM1 transcript is up to 116-fold increased in tumor tissue, with stage III carcinomas showing higher FOXM1 than stage II carcinomas [14]. Work from our group showed that FOXM1 promoted invasiveness and endocrine resistance by expanding the cancer stem-like cell population [38]. Immunohistochemistry staining of tumors from 501 ERα-positive breast cancer patients revealed that high FOXM1 protein was associated with significantly decreased patient survival [38]. In addition, using gene expression data from a large study with Tamoxifen-treated patients with ER-positive breast tumors, we showed that highly elevated FOXM1 mRNA correlated with reduced patient survival [37, 38]. Analysis of tumors from HER2+ patients also showed that FOXM1 expression correlated with poor prognosis in this subtype of breast cancer [44].

FOXM1 is the top-ranked survival-related transcription factor in patients with TNBC, and it is more highly overexpressed in TNBC compared to all other breast cancer subtypes [45, 46]. Consistent with the proliferative phenotype of basal-like TNBC, hyperactivated FOXM1 was found to be a transcriptional driver of the enhanced proliferative signature of basal-like tumors [45]. Immunohistological staining of FOXM1 in tissue microarrays from breast cancer patients correlated with adverse clinicopathological features such as increased tumor size, lymph node metastasis, and higher tumor stage [47]. Notably, brain metastases from breast cancer patients were found to be enriched for cell cycle and G2/M pathways driven by FOXM1 [48]. Thus, FOXM1 overexpression in all breast cancer subtypes make its inhibition a very attractive approach for impeding the progression of all breast cancer subtypes.

## Mechanistic underpinnings of the multiple cancer-promoting roles of FOXM1

Extensive investigation of the molecular mechanisms behind these clinical observations suggests that FOXM1 exerts multiple tumor promoting activities [14, 17]. FOXM1 is upregulated during early cancer development, and contributes to tumorigenesis through stimulation of cell cycle progression via direct proliferation-driving targets like c-Myc [39, 49, 50], and suppression of senescence [20] that can lead to cancer cell survival and relapse. Consistent with this, increasing the expression of FOXM1 in human cancer cells increased their tumorigenicity in xenograft models, while RNAi-mediated knockdown of FOXM1 decreased cancer cell proliferation and suppressed tumor growth in nude mice [51]. Likewise, mouse hepatocytes that are deficient in FOXM1 failed to proliferate and are highly resistant to developing carcinogen-induced liver tumors, while FOXM1-overexpression in LADY and TRAMP prostate cancer models resulted in accelerated development, proliferation, and growth of prostate tumors [52]. The tumorigenic effect of FOXM1 is also driven by other abnormalities frequently found in cancers, such as mutation of important tumor suppressors like Rb and p53, which normally inhibit FOXM1 expression and activity [51].

FOXM1 also stimulates tumorigenesis through regulation of metabolic processes including reprogramming of glucose metabolism and promotion of the Warburg effect [53]. It also enhances angiogenesis through activation of its direct transcriptional target VEGF [54, 55]. FOXM1 is highly involved in inflammation, and promotes tumor formation through direct activation of COX-2 and regulation of macrophage recruitment via its direct transcriptional target CX3CR1 [56, 57]. In primary and metastatic cancers, FOXM1 contributes to invasion, epithelial-mesenchymal transition (EMT) and metastasis through up-regulation of matrix metalloproteinase (MMP)-2, c-Met, pAKT, vimentin, and modulation of E-cadherin [17, 58] and related factors in breast tumors [59]. FOXM1 plays an important role in several aspects of the DNA damage response by regulating the expression of key proteins including XRCC1 and BRCA2 [29]. It is also an integral component of the DNA damage checkpoint signaling network and helps to integrate the DNA damage response with cell cycle progression [60]. When p53 is mutated and FOXM1 is overexpressed, as they are in many cancers, FOXM1 becomes dysregulated after the induction of the DNA damage response and DNA damage checkpoint control is lost [61]. This allows FOXM1 to control the activity of anti-apoptotic genes like Bcl-2 and Survivin and to promote progression of the cell cycle, which supports cancer cell survival through replication defects and DNA damage that drive resistance to some chemotherapeutic drugs [62, 63]. FOXM1 also increases the cancer stem cell population and blocks cell senescence and apoptosis, contributing to the survival, progression, and metastasis of breast cancer [38, 59, 64, 65].

## FOXM1 shares mutual regulatory relationships with ERα and HER2

As noted above, FOXM1 interacts directly with many of the major players and pathways that promote breast cancer growth and progression. Of interest, ERα and FOXM1 have mutual coregulatory actions. ERα controls FOXM1 mRNA and protein levels through direct binding to an estrogen-responsive element at the FOXM1 promoter [66]. Similarly, FOXM1 expression is correlated with ERα expression in many breast cancer cell lines, and overexpression of FOXM1 increases ERα expression at both protein and mRNA levels, augmenting its pro-proliferative effect [43]. Consistent with this, silencing of FOXM1 by siRNA or treatment with FOXM1 small molecule inhibitors abolished estrogen-induced breast cancer cell proliferation [65]. Furthermore, FOXM1 and ERα interact directly, and in a mutually supportive way, through simultaneous binding to many of the same genomic sites in an ERα-dependent manner, and depletion or inhibition of FOXM1 significantly reduces the expression of ERα and ERα-regulated genes [38, 64, 67]. Thus, FOXM1 and ERα share a mutual cancer-promoting regulatory relationship. FOXM1 expression is also highly correlated with HER2 expression in breast cancer [44, 68].

In TNBC, FOXM1 is responsible for driving a cell-cycle enriched gene network that promotes proliferation [65]. In addition, FOXM1 directly regulates MELK, a mitosis-regulating kinase that is essential for mitotic progression in TNBC [69]. FOXM1 also directly regulates expression of eukaryotic elongation factor 2 kinase (eEF2K), a kinase that regulates the ability of eEF2 to mediate TNBC cell proliferation, colony formation, and migration [70]. FOXM1 promotes EMT in breast cancer through direct binding and activation of the SLUG promoter, and it interacts with SMAD3 to sustain TGF-β-induced breast cancer metastasis [71, 72]. In addition, we have shown that FOXM1 increases breast cancer cell aggressiveness, characterized by a migratory and invasive phenotype with modulation of EMT markers like SNAIL, TWIST, CXCR4, and also E-cadherin [38, 59].

## FOXM1 engenders resistance to endocrine therapy and other cancer therapies

**Fig. 2:**
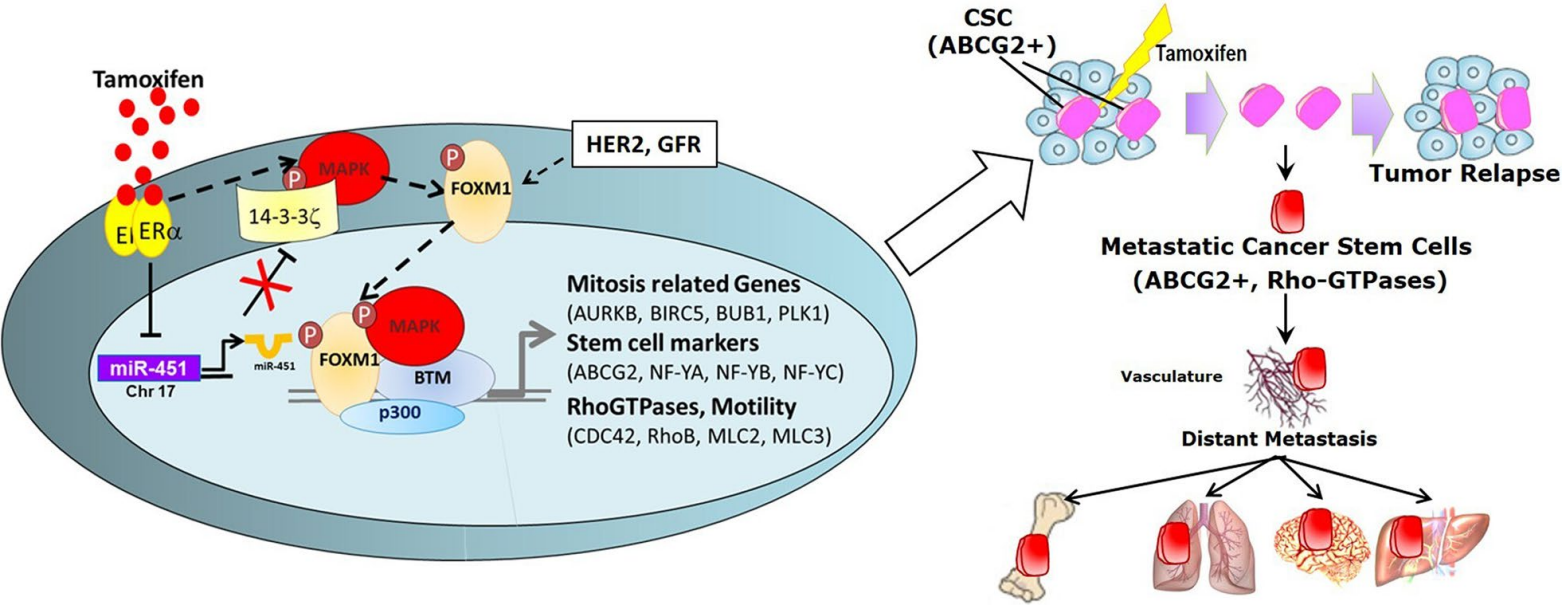
Model showing activities of FOXM1 in breast cancer. FOXM1, in collaboration with the scaffold adaptor protein 14-3-3ζ and protein kinases, increases the Cancer Stem Cell (CSC) population, drives motility, invasiveness and resistance to endocrine and other therapies. Model shows that activation of FOXM1 stimulates its transcriptional activity, enhancing the expression of mitosis related genes, stem cell markers, and RhoGTPases in breast cancer cells, resulting in tumor progression and metastasis to distant sites. Inhibition of FOXM1 activity suppresses tumor growth, invasiveness, and metastasis and reverses drug resistance, as discussed in the text. *BTM*—basal transcriptional machinery, *ERα*—estrogen receptor alpha, *GFR*—growth factor receptor, *MAPK*—mitogen activated protein kinase

FOXM1 promotes the development of endocrine resistance and chemoresistance in breast cancers. Previous work from our group has shown that FOXM1 drives the acquisition of resistance to Tamoxifen in ER-positive breast cancer through expansion of the stem-like cell population and increase in the expression of genes associated with EMT, migration and invasiveness [37, 38] (Fig. 2). FOXM1 was also found to mediate breast cancer cell resistance to HER2-targeting agents, and inhibition of FOXM1 restored sensitization to trastuzumab [73]. Overexpression of constitutively active FOXM1 alone was enough to confer cisplatin resistance in MCF7 cells, and FOXM1 overexpression was also found to confer resistance to paclitaxel by altering microtubule dynamics [15, 62]. Consonant with these observations, siRNA knockdown or pharmacological inhibition of FOXM1 abrogated all of the effects associated with cancer-driving FOXM1 activity, validating FOXM1 as a promising target for suppression of different breast cancers [28, 37, 38, 59, 64, 65].

## Pharmacologic inhibition of FOXM1 and its tumor promoting activity

There is increasing interest in finding small molecule inhibitors of FOXM1 (Table 1). The first reported inhibitors, microbial-derived thiazole antibiotics thiostrepton and siomycin A, are proteasome inhibitors that lower FOXM1 cellular levels and reduce expression of FOXM1-regulated genes. Although thiostrepton was shown to bind directly to FOXM1 [51, 74, 75], its broad inhibitory functions make its FOXM1 selectivity uncertain [76]. As a single agent, thiostrepton had some efficacy on ovarian cancer xenografts when dosed by intraperitoneal (IP) or intravenous (IV) routes [75]. The heterocycle FDI-6, found in a high throughput screen for inhibition of FOXM1-DNA binding [77], is often used as a comparator compound. It has poor in vivo pharmacokinetic (PK) properties [65], although it has activity in TNBC xenografts when dosed IV [78]. A novel cell-penetrating FOXM1-inhibiting p19 tumor suppressor ARF-derived peptide with some D-amino acids to improve its efficiency (p19^ARF^ 26–44 peptide) has been used effectively in treatment of mouse hepatocellular carcinoma and acute lymphoblastic leukemia in vitro and in vivo [79, 80], and in breast cancer studies [38]. Of note, the FOXM1-inhibiting peptide binds to FOXM1 and localizes it to the nucleolus, recapitulating findings with genetic FOXM1 deletion or knockdown.

**Table 1:** Inhibitors of FOXM1

Name	Mechanism	Cancers studied	References
p19^ARF^ 26–44 peptide	Binds, localizes FOXM1 to nucleolus	Breast cancer, HCC, ALL	[38, 79, 80]
Thiozole antibiotics (thiostrepton, siomycin A)	Decrease FOXM1 level	Breast cancer, Ovarian cancer, ALL	[51, 74, 75, 93, 94]
FDI-6	Inhibits FOXM1 DNA binding	Breast cancer	[77]
NB55, NB73, NB115	Bind FOXM1, decrease FOXM1 level	Breast cancer, Ovarian cancer, Multiple myeloma, Melanoma	[28, 53, 59, 64, 65, 75, 81, 91]
STL 427944	Relocates FOXM1 to cytoplasm	Various cancer cells	[82]
DZY-4	Inhibits FOXM1 DNA binding	Ovarian cancer	[83]
FOXM1 PROTACS	Degrade FOXM1	Breast cancer, Liver cancer, Ovarian cancer	[78, 84]

*ALL*—acute lymphoblastic leukemia, *HCC*—hepatocellular carcinoma

We developed FOXM1 inhibitors by optimizing the potency of the best hits we obtained from screening of a large (> 200,000) compound chemical library. Several rounds of structure modifications led to a series of diarylethylene mono and diamine compounds and their methiodide salts, which were FOXM1 inhibitors with desirable PK properties [65]. Studied further were three compounds, NB-55, a monoamine analog with good subcutaneous and oral PK properties, and NB-73 and NB-115, two more potent diammonium salts having very good subcutaneous PK properties [65]. In cell-free assays, these compounds bound to full-length human FOXM1 with submicromolar affinity, and in cell extracts they enhanced FOXM1 proteolytic degradation. They inhibited the growth of several breast cancer cell lines with IC_50_ values as low as 0.5 μM, and they reduced intracellular FOXM1 protein levels [65]. Their patterns of gene regulation were consistent with inhibition of the expression of FOXM1 and FOXM1 target genes, and suppression of signaling pathways promoting cell survival [37, 38, 59, 64, 65]. In breast cancer xenograft models, these compounds effectively suppressed primary tumor growth and metastasis [28, 59, 65]. They also blocked the progression of aggressive multiple myelomas [53] and high grade serous ovarian cancer [75], and melanoma [81], all being cancers with high FOXM1 levels. These compounds also revealed that the coordinated activation of the tyrosine kinase c-Src and FOXM1 drives tumor cell proliferation and breast cancer progression. In a luminal B experimental PyVmT model, c-Src was shown to stimulate the phosphorylation and activation of FOXM1, and targeting FOXM1 with the FOXM1 inhibitors blocked the cell cycle and tumor initiation, as well as tumor progression [28].

The compound STL 427944, recently identified by transcriptomic network analysis, was shown to block FOXM1 activity in various cancer cells by relocating nuclear FOXM1 to the cytoplasm, followed by autophagosomal degradation [82]. DZY-4, identified by virtual screening for compounds that bind to the FOXM1 DNA-binding domain, inhibited the growth of ovarian cancer cells in culture and xenografts when dosed IV [83]. Two FOXM1 proteolysis-targeted chimeras (PROTACs) have been reported recently [78, 84] that degrade FOXM1 and inhibit breast and liver cancer cell proliferation and xenograft growth when dosed IV.

For most inhibitors, even those shown to bind to FOXM1c or FOXM1b, it is not known exactly where they bind to FOXM1 and which isoforms are being targeted, but in most cancers, FOXM1a and FOXM1d are at very low levels, so when total cellular FOXM1 levels are reduced upon treatment with inhibitors or PROTACS, this generally represents largely FOXM1c and FOXM1b. And of the four FOXM1 proteins, FOXM1b and 1c share 98% amino acid identity and are the only transcriptionally active FOXM1 isoforms as discussed in more detail in the previous “FOXM1 Protein and functional domains” section.

## Combination therapies: the need for continuing studies of FOXM1 inhibitors with other drugs to block cancer progression

Combination therapy is now considered a cornerstone of cancer treatment [85, 86]. Despite the growing availability of treatment options, most new targeted drugs have seen little success as single agents in clinical trials [86]. While combination therapy has the potential to reduce side effects and the combination of targeted therapies with endocrine therapies improves outcomes in breast cancer patients, some combinations are associated with higher risk of adverse events [87]. Thus, identifying specific combinations and dosing schedules that provide therapeutic benefits with minimal exacerbation of side effects is an area of need in pre-clinical and clinical studies.

In one of the early successes in breast cancer combination therapy, the addition of the anti-HER2 monoclonal antibody trastuzumab significantly increased the benefits of first-line chemotherapy in patients with HER2-overexpressing metastatic breast cancer [88]. Today, the trastuzumab plus paclitaxel combination is used to treat patients with low-risk HER2-positive tumors, giving excellent long-term outcomes [4]. Between 2015 and 2017, the CDK4/6 inhibitors palbociclib, ribociclib, and abemaciclib received FDA approval for treatment in combination with anti-estrogen therapy of ER(+), HER2(−) metastatic breast cancers [89]. The combination resulted in significant improvement in progression-free survival in these patients. Preclinical studies have demonstrated that CDK4/6 inhibition in combination with anti-estrogen therapy was strongly synergistic, and had the capacity to overcome tamoxifen resistance [85, 90].

The success of combination therapy in hormone receptor-positive and HER2-expressing breast cancers, and the clear need for better therapy options in TNBC, provide a strong rationale for continued study of the use of FOXM1 inhibitors in combination with other drugs. As described earlier, the interaction between FOXM1 and molecular pathways driving tumor growth, metastasis, and treatment resistance provide clear paths for rational design of drug combinations with FOXM1 inhibitors. Indeed, targeting of FOXM1 in combination with chemotherapy has shown benefit in enhanced anti-proliferative and pro-apoptotic effects in several pre-clinical cancer models [91–94].

We recently reported the combination of FOXM1 inhibitors and proteasome inhibitors, such as Bortezomib, to synergistically inhibit proliferation of both ER-positive and triple negative breast cancer [91, 93]. Likewise, we have shown the combined effectiveness of our FOXM1 inhibitors with the CDK4/6 inhibitors palbociclib, ribociclib and abemaciclib in ER-containing breast cancer cells [91]. These findings hold promise for the expansion of translational studies and ultimately clinical trials with agents targeting FOXM1 that may benefit patients with breast cancer and likely other cancers as well.

## Summary and future perspectives

FOXM1 is minimally or not at all expressed in most normal non-regenerating adult tissues but is present and active at all stages of breast cancer development and progression in all subtypes of breast cancer, where it promotes cancer survival, aggressiveness and metastasis. Because FOXM1 is upregulated early during tumorigenesis, as well as generally increasing as cancer stage advances, blocking FOXM1 or its upstream regulators (e.g., c-Src or PLK1) or its downstream targets (e.g., cyclin B1, CENP-F, Aurora Kinase B, or others) could have a profound beneficial impact on the disease by arresting cancer at an early stage as well as complementing and enhancing the effectiveness of other drug treatments. FOXM1 suppression might also resensitize breast cancer cells to cancer drugs that were previously useful, and thereby overcome acquired drug resistance (e.g., to tamoxifen or trastuzumab, or chemotherapy). Recently developed small molecule inhibitors of FOXM1 have been shown to suppress hormone receptor-positive luminal A and luminal B breast cancers and triple negative breast cancers [59, 64, 65, 77], and to block the coordinated activation of c-Src and FOXM1 that drives tumorigenesis and breast cancer progression [28]. Response to FOXM1 is also highly dependent on cell context because FOXM1 has numerous interacting partners (Rb, p53, PLK1, etc.) whose level and state (high, low, mutated) may vary in different cells and under different treatment conditions.

Preclinical studies support the value of future directions involving novel FOXM1 inhibitors in combination with CDK4/6 inhibitors, proteasome inhibitors, and other classes of cancer drugs as new treatment modalities to overcome resistance to other therapeutic drugs. In adult differentiated tissues, however, we have to keep in mind that, although FOXM1 is usually very low, the Human Protein Atlas shows FOXM1 to be expressed in rapidly proliferating tissues such as testis, thymus and bone marrow, although at levels considerably lower than in cancer cells. Likewise, its presence and proliferative activity can be activated during regenerative processes after injury in tissues such as liver and lung [18, 95]. Hence, the potential side effects of blockade of FOXM1 activity during cancer treatment are likely to be limited in otherwise healthy individuals but might interfere with the function of some tissues and the regeneration or healing of injured tissues [96]. Collectively, the approaches discussed in this Review will hopefully continue to be studied with the goal of improving outcomes and overall survival of patients with breast cancer and potentially benefiting patients with other types of cancers as well.

## Data Availability

Not applicable in this review article.

## Abbreviations

AURKB: Aurora kinase B
CCN: Cyclin
CDK: Cyclin dependent kinase
CENP-F: Centromere protein-F
DBD: DNA binding domain
eEF2K: Eukaryotic elongation factor 2 kinase
EMT: Epithelial mesenchymal transition
ER: Estrogen receptor
FOXM1: Forkhead box M1
HER2: Human epidermal growth factor receptor 2
MAPK: Mitogen activated protein kinase
MMP: Matrix metalloproteinase
NRD: Negative regulatory domain
PK: Pharmacokinetic
PLK1: Polo-like kinase 1
PROTACs: Proteolysis-targeted chimeras
TAD: Transcriptional activation domain
TNBC: Triple negative breast cancer
